# Detachment of Hexokinase II From Mitochondria Promotes Collateral Sensitivity in Multidrug Resistant Chronic Myeloid Leukemia Cells

**DOI:** 10.3389/fonc.2022.852985

**Published:** 2022-05-26

**Authors:** Thaís Oliveira, Douglas Lemos, Louise Jean, Jéssica M. Kawashima, Vitória R. de Azevedo, Eduardo J. Salustiano, Vivian M. Rumjanek, Robson Q. Monteiro

**Affiliations:** ^1^Laboratório de Trombose e Câncer, Instituto de Bioquímica Médica Leopoldo de Meis, Universidade Federal do Rio de Janeiro, Rio de Janeiro, Brazil; ^2^Laboratório de Imunologia Tumoral, Instituto de Bioquímica Médica Leopoldo de Meis, Universidade Federal do Rio de Janeiro, Rio de Janeiro, Brazil

**Keywords:** hexokinase II, glutathione, chemoresistance, chronic myelogenous leukemia, metabolism

## Abstract

Chronic Myeloid Leukemia is a neoplastic disease characterized by the abnormal expansion of hematopoietic cells with compromised functions. Leukemic cells often display a multidrug resistance phenotype, enabling them to evade a number of structurally unrelated cytotoxic compounds. One of those mechanisms relies on the high expression of efflux transporters, such as the ABC proteins, whose activity depends on the hydrolysis of ATP to reduce intracellular drug accumulation. In the present work, we employed a well-known erythroleukemia cell line, K562, and a multidrug resistant derivative cell, FEPS, to evaluate how hexokinase II, a key regulator for the rate-limiting step glycolysis, contributes to the establishment of the multidrug resistance phenotype. We found that multidrug resistant cells primarily resort to glycolysis to generate ATP. Clotrimazole reduced the expression of mitochondrial hexokinase II, which destabilized bioenergetic parameters such as reactive oxygen species production, ATP, and glutathione levels on multidrug resistant cells. This impaired the activity of ABCC1, leading to increased drug accumulation and cell death. In summary, we propose that decoupling of hexokinase II from the mitochondria emerges as a promising strategy to generate collateral sensitivity and aid in the management of chronic myeloid leukemia in chemotherapy-refractory patients.

## 1 Introduction

Chronic Myeloid Leukemia (CML) is a neoplastic disease characterized by the reciprocal translocation between the long arms of chromosomes 9 and 22, which results in the expression of the chimeric oncoprotein BCR-ABL. This is a constitutively active tyrosine kinase that regulates various signalling pathways that promote the abnormal expansion of hematopoietic cells and impairment of their functions (1, 2). Mutations on BCR-ABL are the most frequent cause of treatment failure with specific inhibitors such as imatinib (3), which is frequently employed in combination with standard chemotherapeutic drugs for inducing remission (4).

The stress caused by a variety of cytotoxic compounds might select neoplastic cells with enabling characteristics to evade cell death by chemotherapeutic drugs with distinct mechanisms of action, a phenotype of multidrug resistance (MDR). The main features of this phenotype are the increase in expression of antiapoptotic proteins, the activation of DNA damage repair mechanisms, drug sequestration and/or reduction of intracellular drug accumulation (2–6). Increased expression and activity of membrane efflux transporters have been linked to unsuccessful outcomes in CML (7). Overall, 30-40% of CML patients exhibit some degree of resistance to imatinib, and more potent drugs present little effect to overcome multifactorial resistance (8). As such, treatment options are still in demand for those patients.

ABCB1 is an active transporter which interacts with diverse hydrophobic, neutral, or positively charged substrates, including chemotherapeutic drugs such as DNR, vincristine (VCR), vinblastine, paclitaxel, among others (9). ABCC1, in turn, shows higher affinity for negatively charged molecules (10). Our group recently reviewed the relevance of ABCB1 for CML patients, highlighting polymorphisms, associations with microRNAs and inhibitor of apoptosis proteins (IAPs) that relate to therapeutic resistance. In addition, we observed increased expression, mRNA and efflux activity among five different CML cohorts (8). The impact of ABCC1 for CML is less clear in the clinic (11–13); nevertheless, in a small group of CML treatment-refractory patients, we described increased expression and activity of ABCC1 in 11 and ABCB1 in 12 out of 13 individuals (14, 15).

The overexpression of active efflux transporters in MDR cells represents a high energetic demand, which inevitably associates the processes that regulate the emergence of this phenotype to intermediates of various metabolic pathways. Thus, glycolysis appears as an essential energy source. One of the fundamental elements in this process is hexokinase (HK), the first enzyme in the glycolytic pathway, whose main function is to phosphorylate glucose to glucose-6-phosphate (G6P), allowing its influx into glycolysis or feeding the pentose phosphate pathway (PPP) (16). Furthermore, by providing the PPP with G6P, HK contributes to the recycling of glutathione to its reduced form (GSH), which is critical for cells to maintain redox balance (16). HK has been described as a moonlighting protein, able to perform functions other than the catalysis of metabolic reactions (17, 18). Therefore, since its mitochondrial-bound state has direct access to mitochondrial ATP, it may use it preferentially to phosphorylate glucose, stimulating ATP production and increasing the efficiency of the electron transport system (ETS), thereby reducing the generation of reactive oxygen species (ROS) (19–21). Another critical feature of mitochondrial HK is its interaction with the voltage-dependent anion channel (VDAC), which blocks the activation of the apoptotic pathway (22, 23).

In order to better understand this phenomenon, we employed an experimental model of CML, which mimics the MDR phenotype using the cell lines K562 and FEPS. K562 derives from the pleural effusion of a terminally ill patient with highly undifferentiated CML (24), whereas FEPS was selected from K562 through exposure to increasing doses of daunorubicin (DNR), a pro-oxidant anthracycline which stabilizes the DNA topoisomerase II complex preventing correct replication. Among diverse altered pathways (25), this selection culminated in the increased expression and activity of the transporters P-glycoprotein/ATP binding cassette subfamily B member 1 (Pgp/ABCB1) and multidrug resistance-associated protein 1/ATP binding cassette subfamily C member 1 (MRP1/ABCC1), which confers FEPS a greater chemoresistance profile when compared to their parental counterpart (26, 27).

In the present work, we employed K562 and FEPS CML cell lines to evaluate the possible metabolic disparities caused by the removal of HKII from the mitochondrial membrane and how such perturbations affect the MDR phenotype in CML.

## 2 Materials and Methods

### 2.1 Cell Lines

The CML lines K562 and FEPS (K562/DNR), were grown in RPMI-1640 medium (Sigma-Aldrich) supplemented with 10% fetal bovine serum (FBS) at 37 °C and 5% CO_2_. FEPS is a stable K562-derived cell line established upon exposure to increasing doses of DNR (26). FEPS cell line was cultivated in the presence of 500 nM of DNR to maintain its resistant profile up to 72 h prior to carrying out the experiments. Mycoplasma contamination was routinely tested by PCR using primers described elsewhere (28).

### 2.2 High-Resolution Respirometry

Assessment of oxygen consumption was performed on a high-resolution respirometry system (OROBOROS Instruments – Oxygraph2k – Innsbruck, Austria). 2×10^6^ intact cells were incubated in Dulbecco’s Modified Eagle’s Medium without glucose, L-glutamine, phenol red, sodium pyruvate and sodium bicarbonate supplemented with 11 mM glucose and 2 mM glutamine (equivalent concentrations to RPMI-1640 culture medium). After the respiration stabilization period, the following modulators of mitochondrial function were added: oligomycin (1 µg/mL), carbonyl cyanide-4-(trifluoromethoxy) phenylhydrazine - FCCP (50 nM), rotenone (0.5 µM) and antimycin A (2 µg/mL). From the addition of these modulators, it is possible to evaluate the following respiratory parameters: (i) routine oxygen flow – in absence of modulators; (ii) oxygen flux uncoupled from ATP synthesis – through treatment with oligomycin, ATP synthase inhibitor; (iii) oxygen flux coupled to ATP synthesis – by subtracting the routine flux values ​​from those obtained after the addition of oligomycin; (iv) maximum capacity of the ETS – due to the acceleration of the electron transport necessary for the maintenance of the membrane potential resulting from FCCP treatment, which dissipates the proton gradient; (v) residual oxygen flow – through the inhibition of complexes I and III by treatment with rotenone and antimycin A, respectively, which makes it possible to measure the oxygen flow not related to mitochondrial functions; (vi) reserve capacity – by subtracting the routine flow values ​​from those obtained after adding FCCP (maximum capacity). To acquire the graphs and analyze the values, the DatLab version 5.1 program (OROBOROS Instruments – Oxygraph2k) was used.

### 2.3 Lactate Quantification Assay

For lactate release, 4×10^5^ cells per condition were incubated for 24 h at 37°C and 5% CO_2_ in DMEM without phenol red and FBS supplemented as described before (11 mM glucose and 2 mM glutamine) in a reaction buffer pH 9.2 containing 0,6 M glycine, 0,35 M hydrazine and 15 mM β-NAD^+^. The lactate was quantified using the formation of nicotinamide adenine dinucleotide (NADH) and pyruvate in the presence of excess NAD^+^. The reaction was catalyzed by the addition of lactate dehydrogenase (LDH) (2 U/mL), and the concentration of lactate was calculated from the extinction coefficient of NADH (6,22 × mM^-1^ × cm^-1^) in the Spectramax spectrophotometer M4 Multi-Mode Microplate Reader at 340 nm.

### 2.4 ATP Quantification Assay

ATP content was measured from 10^5^ cells following the protocol guidelines of the ATP Determination kit (Thermo Fisher). This assay measures the luminescence of the reaction, based on the luciferin-luciferase system, in a dark 96-well plate. Cells were placed in the presence of reaction medium (1 mM 1,4-dithiothreitol (DTT), 20× Reaction Buffer, 0.5 mM luciferin and 1.25 mg/ml luciferase) at the time of reading. The bioluminescence generated, directly proportional to the ATP content, was measured by spectrophotometry in the device’s default condition. The ATP was quantified from the correlation to the values obtained in the standard curve.

### 2.5 RNA Extraction and cDNA Synthesis

Total RNA was isolated from cells using TRIzol reagent (Invitrogen) following the manufacturer’s instructions. Total RNA was quantified spectrophotometrically. 1 µg RNA was treated with 1 U of RNAse-free DNAse for 30 minutes at 37 °C. Reactions were stopped by adding 1 µL of ethylenediamine tetraacetic acid (EDTA) 20 mM and heating for 10 minutes at 65 °C. cDNA synthesis was performed using the DNAse treated RNA using the High-Capacity cDNA Reverse Transcription Kit from Applied Biosystems in accordance with the manufacturer’s instructions.

### 2.6 Real-Time PCR

Gene expression analysis was performed using 7500 Real-Time PCR (Applied Biosystems) and power SYBR-GREEN PCR master MIX (Applied Biosystems). For this test primer pairs were synthesized based on GenBank sequences of mRNA. The comparative Ct method was used to measure changes in gene expression levels (29). Actin was employed as an endogenous control.

### 2.7 Mitochondria Preparations

10^6^ cells were lysed during 3 minutes in a Potter-Elvehjem homogenizer in ice, in a buffer containing 10 mM Tris-HCl pH 7.4, 0.25 M sucrose, 20 mM sodium fluoride (NaF) and 5 mM EDTA (cell lysis buffer). The homogenates were centrifuged for 5 minutes at 4°C at 1000×g. The supernatant was collected and centrifuged for 15 minutes at 4°C at 10000×g. The supernatant (cytosolic fraction) was set aside, and the pellet (mitochondrial fraction) suspended with the cell lysis buffer. Protein concentration was assayed using the Bradford method.

### 2.8 Western Blotting

Cell pellets were lysed as described in mitochondrial preparations. 40 µg of protein extracts were fractionated by standard 10% SDS-PAGE and transferred to nitrocellulose membranes by electro blotting in a buffer consisting of 39 mM glycine, 48 mM Tris-base, sodium dodecyl sulfate (SDS) 0.037% (w/v) and methanol 20% (v/v). Proteins were detected using primary antibodies diluted in Tris-buffered saline (TBS), Tween 20 0.1% (v/v) and bovine serum albumin (BSA) 5% (w/v). Rabbit anti-human HKII and anti-G6PDH polyclonal primary antibodies were obtained from Cell Signaling Technology^®^. Rabbit anti-human GR primary antibody was obtained from Abcam^®^. The secondary antibody for all incubations was the IRDye 680RD goat anti-rabbit immunoglobulin. β-actin was used as loading control because its expression not being altered between K562 and FEPS (25). Bands were visualized in a Li-Cor Odyssey Western blot imaging.

### 2.9 Enzymatic Activities

#### 2.9.1 Hexokinase II

The assay method coupled to glucose-6-phosphate dehydrogenase (G6PDH) purified from the bacteria *Leuconostoc mesenteroides*, which is capable of using NAD^+^ as an electron receptor, was employed to evaluate the enzymatic activity of HKII. This enzyme oxidizes the reaction product of HKII (G6P) at the expense of NAD^+^ generating NADH. The production of NADH is measured by spectrophotometry and the specific activity of the enzyme is obtained from the molar extinction coefficient of NADH at 340 nm (ϵ 340 nm = 6.22 × 10^-1^ mM × cm^-1^). The following is the stoichiometric relationship where 1 mol of G6P produced is equivalent to 1 mol of NAD^+^ reduced. For the assay, 60 µg of protein were used under the conditions tested. The reaction used contained 50 mM Tris-HCl pH 8.0, 10 mM magnesium chloride (MgCl_2_), 2 mM ATP, 0.1 mM Triton X-100, 5 mM glucose, 1 mM β-NAD^+^ and 1 U/mL G6PDH at 37°C for 20 minutes. The experimental points for HK activity were fitted to Michaelis–Menten and Lineweaver-Burk equations to calculate the Vmax and Km for HK plots.

#### 2.9.2 Glucose-6-Phosphate Dehydrogenase

G6PDH catalyzes de conversion of G6P to 6-phosphoglucolactone producing NADPH, which is hydrolyzed to 6-phosphogluconate (substrate for 6-phosphogluconate dehydrogenase) producing NADPH as well, G6PDH activity was calculated by subtracting the activity of 6PGDH from total enzyme activity. To obtain the total dehydrogenase activity, substrates for both dehydrogenase enzymes were added to a cuvette. In another cuvette, substrates for the second enzyme, 6PGDH, were added to obtain the rate of this enzyme. The specific enzyme activity is calculated using the molar extinction coefficient of NADPH at 340 nm (ϵ 340 nm = 6.22 × 10^-1^ mM × cm^-1^). The following is the stoichiometric relationship where 1 mol of 6-phosphoglucone-δ-lactone produced is equivalent to 1 mol of NADPH. For the assay, 60 µg of protein referring to the cytosolic fraction of the cells were used under the conditions tested. For total enzyme activity the proteins were kept with reaction medium containing 50 mM Tris-HCl pH 8.0, 0.5 mM NADP, 0.1 mM Triton X-100, 2 mM G6P and 5 mM MgCl_2_. In the 6PDH assay, substrates’ concentrations were 50 mM Tris-HCl pH 8.0, 0.2 mM 6PG and 0.1 mM NADP^+^. The activity assay was performed at 37°C for 10 minutes.

#### 2.9.3 Glutathione Reductase

GR reduces the oxidized form of glutathione (GSSG) to GSH, using NADPH as an electron acceptor and converting it to NADP^+^. Thus, GR activity is measured by reducing the absorbance using 100 µg of protein, which reflects the consumption of NADPH in the reaction. They were kept with the reaction medium containing 100 mM potassium phosphate buffer pH 7.5, 1 mM EDTA, 2 mM GSSG, 2 mM NADPH and water at 25°C for 10 minutes. The colorimetric products formed after all enzymatic activity assays, respecting the particularities of each, were quantified by spectrophotometry at 340 nm.

### 2.10 Treatments

Clotrimazole (Sigma-Aldrich), an imidazole derivative, was dissolved in dimethylsulfoxide (DMSO) to a stock concentration of 2.9 mM which was used for cell treatments at the final concentrations of 15 µM, 35 µM and 50 µM for 1, 3, 6 and 24 h. The previous incubation of cells with this compound was employed to induce detachment of HKII from the outer mitochondrial membrane, since this effect is already well described in the literature (30–32). The cells were treated with 11 mM 2-deoxy-D-glucose (2DG) – equimolar to the concentration of glucose present in the RPMI-1640 medium – for 72 h in combination with 500 nM DNR or 60 nM VCR (VCR). Alternatively, cells were previously incubated for 2 h with 1 mM NAC before the assays.

### 2.11 Cell Viability

Cell viability was assayed by crystal violet and cell count with trypan blue. Crystal violet interacts with viable cell DNA while trypan blue permeates and stains only dead cells. For the crystal violet assay, 2×10^4^ cells were fixed with 4% paraformaldehyde after treatment under the tested conditions and stained with 0.05% crystal violet for 30 minutes under agitation and protected from light. Then, the wells were washed with deionized water to remove excess dye and the crystals were solubilized with 70% ethanol. Intensity of staining, directly proportional to the percentage of viable cells, was quantified by spectrophotometry at 570 nm. Trypan blue was employed at 0.08% in a Neubauer chamber for counting under an optical microscope after treatment.

### 2.12 MitoSOX

The mitochondrial superoxide $\left({\text{O}}_{2}^{-}\right)$ was measured from 10^5^ cells treated as described before, following the protocol of the reagent MitoSOX™ Red (Thermo Fisher). MitoSOX™ Red is a fluorogenic dye specifically targeted to mitochondria in live cells. Oxidation of MitoSOX™ Red reagent by superoxide produces fluorescence, selectively targeting mitochondria of live cells. It is rapidly oxidized by superoxide generating a fluorescent product but not by other ROS and reactive nitrogen species. The oxidized product is highly fluorescent upon binding to nucleic acid. Maximum absorption/emission: ∼510/580 nm.

### 2.13 Lipid Peroxidation

Free radicals react with membrane lipids and cause peroxidation with the consequent formation of malondialdehyde (MDA). Following heating in the presence of thiobarbituric acid (TBA), the reaction forms a fluorescent compound that is quantified in a fluorimeter (excitation: 515 nm; emission: 553 nm) (33). For this assay, 1×10^6^ cells were macerated in phosphate buffer for protein extraction. Proteins were quantified for later normalization of results and part of the obtained sample was incubated with 20% trichloroacetic acid (TCA) and 0.67% TBA (in sodium sulfate) in a boiling bath for 2 h. Then, n-butanol was added and the samples were centrifuged for 3 minutes at 5000×g and the fluorescent product, present in the upper fraction, was quantified by spectrophotometry under excitation of 515 nm and emission of 553 nm. A standard curve with 10× diluted tetramethoxypropane (TMP) was used as reference for the quantification.

### 2.14 Mitochondrial Reducing Capacity Analysis

Mitochondrial reducing capacity was measured by colorimetric assay with MTT (3-(4,5-dimethylthiazol-2-yl)-2,5-diphenyltetrazol bromide) after a 1 h incubation with 15 µM, 35 µM and 50 µM CTZ. The MTT solution was prepared at a concentration of 5 mg/mL diluted in 1× phosphate buffered saline (PBS). MTT reacts with mitochondrial dehydrogenases reducing tetrazolium salts, resulting in blue-purple formazan crystals. Those were diluted in DMSO and the absorbance, directly proportional to the number of formed crystals, was measured by spectrophotometry at 492 nm.

### 2.15 GSH Quantification

Glutathione levels were measured in accordance with the protocol described by Biswas et al. (34). Glutathione is a key antioxidant tripeptide (L-γ-glutamyl-L-cysteinyl-glycine) that represents the major non-protein thiol in many organisms. Within cells, glutathione exists in two different forms: the reduced form GSH and GSSG, the oxidized form. Oxidative stress has a profound effect on the cellular thiol balance and can lead to a decreased GSH/GSSG ratio. ROS - superoxide anion is neutralized by GSH through a cascade of detoxification mechanisms involving enzymes such as GR. The colorimetric reaction generates a product directly proportional to levels of glutathione, which was quantified by spectrophotometry at 415 nm.

### 2.16 TUNEL Assay

Apoptotic cells were quantified using the Click-iT^®^ TUNEL Alexa Fluor^®^ imaging assay kit from 2×10^5^ cells, following the protocol guidelines. TUNEL assay is based on the incorporation of modified uridine triphosphate (dUTPs) by the enzyme terminal deoxynucleotidyl transferase (TdT) at the 3’-OH ends of fragmented DNA, a hallmark as well as the ultimate determinate of apoptosis. The Click-iT^®^ TUNEL Alexa Fluor^®^ imaging assays utilize a dUTP modified with an alkyne, a small, bio-orthogonal functional group that enables the nucleotide to be more readily incorporated by TdT than other modified nucleotides. Detection is based on a click reaction, 3-6 a copper-catalyzed reaction between an azide and alkyne. The fluorescence generated is directly proportional to the number of apoptotic cells and was quantified by spectrophotometry under excitation of 495 nm and emission of 519 nm.

### 2.17 ABC-Mediated Efflux Assays

The ABCB1 and ABCC1 transport assays were performed, respectively, with the use of the Rho 123 and 5(6)-carboxyfluorescein diacetate (CFDA) dyes (both from Sigma-Aldrich) as previously described (27). Rho 123 and CFDA passively distribute into the cell, and while the first is fluorescent and is actively extruded by ABCB1, the latter undergoes hydrolysis by nonspecific esterases in the cytosol, originates the fluorescent substrate carboxyfluorescein (CF) that only then is transported out by ABCC subfamily members, notably ABCC1 (35). Briefly, assays were performed in two 30-minute steps, sufficient for the accumulation and efflux of dyes. Both steps were carried on at 37 °C in 5% CO_2_ in a light-protected environment. First, 2×10^4^ cells/mL were treated for 1 h with 35 mM CTZ as described prior to the assays. Then, 2×10^5^ FEPS cells were incubated in 96-well plates with 250 nM Rho 123 or 500 nM CFDA in fresh RPMI medium to allow accumulation of the dyes within cells (accumulation phase). Following, cells were centrifuged at 200×g for 7 minutes and then resuspended in fresh RPMI to allow efflux of the dyes (efflux phase). Inhibition of ABCB1 efflux was performed in the presence of the calcium channel blocker verapamil (VP) (36), and the quinoline derivative MK-571 was employed to inhibit ABCC efflux (37) (inhibited efflux). As negative control, cells were exposed to medium only (free efflux). Then, cells were again centrifuged, resuspended in cold PBS and maintained on ice until acquisition by flow cytometry.

### 2.18 Assessment of DNR Uptake

Assays were performed in similar conditions to the ABC-mediated efflux assays. FEPS cells were treated with CTZ as described, and then incubated in 96-well plates with 2.5 or 5.0 μM DNR for 1 h. In parallel, cells were incubated with the ABCB1 inhibitor VP, the ABCC inhibitor MK-571 or with reduced glutathione. As negative control, cells were exposed to medium only, and treated as described before acquisition by flow cytometry.

### 2.19 Flow Cytometry

The median fluorescence intensities (MFI) from 10^4^ viable cells, gated in accordance with forward and side scatter parameters representative of cell size and granularity, were acquired using the FL1-H filter (for Rho 123 and CF) or the FL3-H filter (for DNR) on a BD FACS Calibur flow cytometer (BD Biosciences, San Jose, CA, USA) and represented on histograms. All post-analyses were performed on Summit version 4.3 software (Dako Colorado, Inc., Fort Collins, CO, USA).

### 2.20 Statistical Analysis

Statistical analysis was performed using GraphPad Prism 8 (GraphPad Software, inc.). Results are expressed as mean + SEM values for an independent experiment. Comparisons between groups were done by one-way ANOVA and *a posteriori* Dunnett’s test. When appropriate, unpaired Student’s t-tests or Mann-Whitney’s test were employed. Differences of p < 0.05 were considered significant.

## 3 Results

### 3.1 MDR CML Cells Show Lower Oxygen Flow Than Sensitive Cells

**Figure 1:**
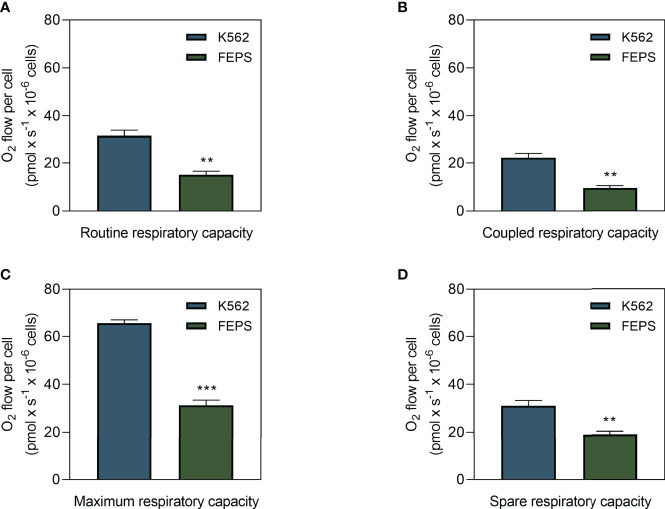
Oxygen flux in drug-sensitive and MDR CML cells. K562 and FEPS cells were cultured as described in Materials and Methods, and the amount of oxygen consumed by a total of 2×10^6^ cells was evaluated. **(A)** Routine oxygen flow on CML cells. **(B)** Coupled respiratory capacity of CML cells. **(C)** Maximum respiratory capacity of CML cells in response to modulation of the ETS with 50 nM FCCP. **(D)** Spare respiratory capacity = maximum capacity – basal oxygen flow. Bars represent the mean oxygen flow + SEM in pmol × s^-1^ normalized to the total cell count from three independent experiments. K562 (blue bar), FEPS (green bar). (**) p < 0.01; (***) p < 0.001.

Metabolism comprises a series of sequential regulated reactions to obtain energy or transform it, that can be produced through oxidative phosphorylation, coupled with oxygen consumption or as occurs in glycolysis (19, 21). Some tumors preferentially use one of these pathways to supply their energy demands depending on their metabolic profiles (16). One way to measure the mitochondrial contribution to these processes is through the quantification of oxygen flow. As shown in **Figure 1**, FEPS exhibited significantly lower basal oxygen flow (**Figure 1A**), coupled respiratory capacity (**Figure 1B**), maximal and spare respiratory capacities (**Figures 1C, D**) than K562. Basal oxygen flow represents the routine oxygen demand without any interference. Coupled respiratory capacity refers to mitochondrial oxygen consumption that is coupled to ATP synthesis. The maximum capacity is experimentally induced by titration with uncouplers, such as FCCP, to collapse the proton gradient across the inner mitochondrial membrane to measure the capacity of the ETS. Spare respiratory capacity reflects the extent of the mitochondria response to stressful situations or increased energy demand, essential for cell survival. These results suggest that adaptation to chemotherapeutic stress on FEPS cells reduced the oxidative metabolism compared to the parental K562.

### 3.2 Maintenance of the MDR Phenotype Demands a High Energy Input Accomplished Through Glycolysis

**Figure 2:**
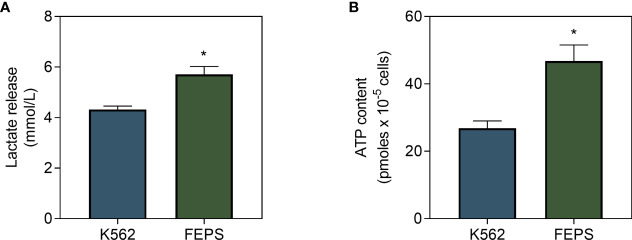
Lactate release and ATP content in drug-sensitive and MDR CML cells. K562 and FEPS cells were cultured as described in Materials and Methods. The amount of lactate released by a total of 4×10^5^ cells and ATP content of a total of 10^5^ cells were evaluated. **(A)** Indirect quantification of lactate released after 24 h through the formation of NAD^+^ from NADH. Data express the mean lactate release + SEM in mmol/L. **(B)** ATP content in accordance with the light emitted after reaction with luciferase. Data express ATP quantification mean + SEM in picomoles. Bars represent data from three independent experiments + SEM. K562 (blue bar), FEPS (green bar). (*) p < 0.05.

Poorly oxidative cells preferentially resort to the glycolytic pathway to obtain energy. In this context, pyruvate is converted to lactate instead of feeding oxidative phosphorylation through the Krebs cycle (19). Results on **Figure 2** show that FEPS released significantly more lactate than K562 (**Figure 2A**), supporting the hypothesis that MDR cells preferentially engage in glycolysis in detriment of the oxidative pathway. Nevertheless, cells need sufficient energy input to supply basic cellular processes, and the primary way to store it is through ATP production. **Figure 2B** showed that the ATP content of FEPS was approximately twice as that measured in K562. Considering the lower mitochondrial oxygen flux (**Figure 1**) associated with higher lactate release (**Figure 2A**), data suggest that MDR cells preferentially utilize the glycolytic pathway to increase ATP input.

### 3.3 MDR CML Cells Present Higher HKII Expression and Activity

**Figure 3:**
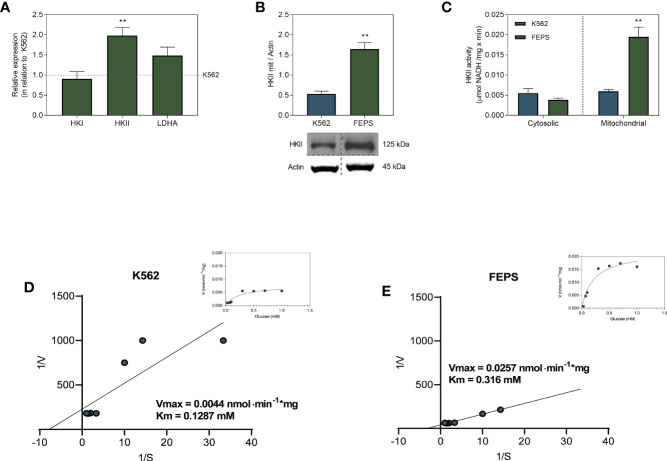
Expression and activity of enzymes from the glycolytic pathway. K562 and FEPS cells were cultured as described in Materials and Methods. 1 µg of RNA was used to synthesize the cDNA, 40 µg of protein for Western blotting, and 60 µg of protein to enzymatic activity. **(A)** Mean expression of HKI, HKII, and LDHA genes in FEPS by real-time PCR in relation to K562 (dotted blue horizontal line). **(B)** Expression of HKII was evaluated by Western blotting as described in Materials and Methods. Densitometric analyses were calculated in relation to the expression of actin for each cell. **(C)** HKII activity was measured through the conversion of NAD^+^ to NADH by G6PDH, considering the stoichiometric ratio where 1 mol of G6P produced is equivalent to 1 mol of reduced NAD^+^ in µmol NADH/mg × min. **(D, E)** The experimental points for HK activity were fitted to Lineweaver-Burk and Michaelis–Menten (inset) equations to measure Vmax and Km for HK isoforms. Bars represent the mean + SEM of three independent experiments. K562 (blue bar/circles), FEPS (green bar/circles). (**) p < 0.01.

Hexokinase is the first enzyme of the glycolytic pathway; it is present in four isoforms (HKI-IV) and its main function is to convert glucose into G6P. Pyruvate generated at the end of glycolysis enters the Krebs cycle and fuels oxidative phosphorylation or is converted to lactate by LDH (16). Among its isoforms, only HKI and HKII have the ability to bind to the mitochondrial membrane (21, 38). Despite sharing this feature, they may be differentially expressed in a tissue-specific manner (39, 40). **Figure 3A** shows that although there are no significant differences in HKI and LDHA gene expression between FEPS and K562, HKII expression is approximately two-fold higher in MDR cells. An increase of HKII at gene level in FEPS was accompanied by increased expression of this enzyme in the mitochondrial fraction, where levels three times higher than those observed in K562 were detected (**Figure 3B**). Accordingly, the enzymatic activity of HKII on the mitochondrial fraction was four times higher in FEPS as compared to K562 (**Figure 3C**). Michaelis-Menten and Lineweaver-Burk plots (**Figures 3D, E**) confirm that the hexokinase isoform II is the most active. K562 and FEPS affinities for glucose were 0.1297 mM and 0.316 mM, similar to the Km values reported for HKII of 0.13-0.3 mM (39, 41, 42). The presence of HKII mostly in the mitochondrial fraction (**Figure 3B**) might relate to the high ATP content of FEPS (**Figure 2B**), as it can directly use and optimize its production by increasing the efficiency of ETS.

### 3.4 MDR CML Cells Exhibit Low Levels of Oxidative Markers and High Sensitivity to 2-Deoxy-D-Glucose

**Figure 4:**
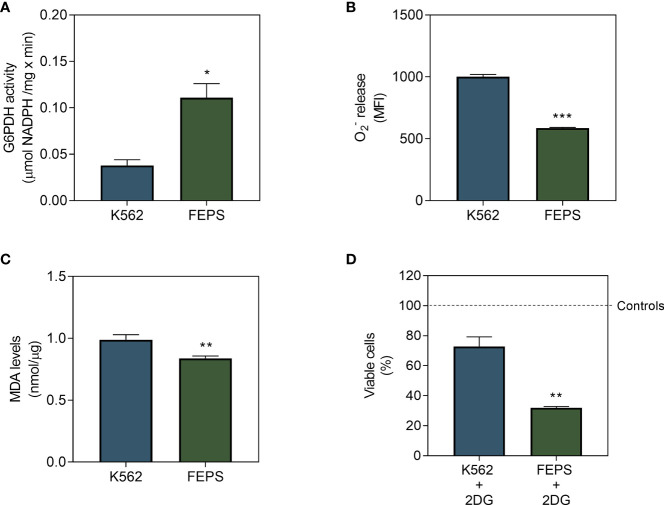
Effect of 2DG on G6PDH, on markers of mitochondrial damage and on the viability of CML cells. K562 and FEPS cells were cultured as described in Materials and Methods. 60 µg of protein was used to quantify G6PDH activity, a total of 10^5^ cells was used to measure ${\text{O}}_{2}^{-}$ release, a total of 10^6^ cells was used to measure MDA levels and 2×10^4^ cells were used to viability assay. **(A)** G6PDH activity measured through the conversion of NADP^+^ to NADPH resulting from the oxidation of G6P to 6-phosphoglucone-δ-lactone, considering the stoichiometric ratio of 1 mol of 6-phosphoglycon-δ-lactone to 1 mol of NADPH as quantified by absorbance (340 nm). Data represent the mean of G6PDH activity + SEM in µmol NADPH/mg × min. **(B)** Superoxide release quantified by MitoSOX. Data represent the mean fluorescence intensity + SEM produced after the oxidation of the reagent by mitochondrial ${\text{O}}_{2}^{-}$ **(C)** MDA produced after the reaction of free radicals with the mitochondrial membrane. Data represent the mean MDA + SEM released in nmol/µg of protein. **(D)** Cell viability after 24 h in the presence of 2DG by crystal violet, represented by the percentage of viable cells mean after treatment in relation to control condition for each cell. Bars represent the mean + SEM obtained from three independent experiments. K562 (blue bar), FEPS (green bar). (*) p < 0.05; (**) p < 0.01; (***) p < 0.001.

HK stimulate the PPP when associated with the outer mitochondrial membrane by providing G6P to the first enzyme of this pathway, G6PDH. This coenzyme, in turn, maintains adequate levels of NADPH and glutathione to protect cells from the oxidative damage caused by ROS (16). As depicted in **Figure 4A**, FEPS presented significantly increased G6PDH activity relative to K562. High activity of this enzyme may contribute to a reduction of ${\text{O}}_{2}^{-}$ (**Figure 4B**) and MDA (**Figure 4C**) in MDR cells.

Lipoperoxidation occurs when ROS, namely the superoxide anion, react with phospholipids of the inner mitochondrial membrane, often resulting in cell death due to the accumulation of highly reactive compounds such as the unsaturated aldehyde MDA (33). Impairment of the PPP upon treatment with 2DG further reduced the viability of MDR cells compared to the sensitive ones (**Figure 4D**). Together with the observed increase in HKII activity (**Figure 3C**), this suggests that the PPP might play an important role for the viability of drug-resistant cells.

### 3.5 Clotrimazole Decreases Mitochondrial HKII Activity and Expression in MDR Cells

**Figure 5:**
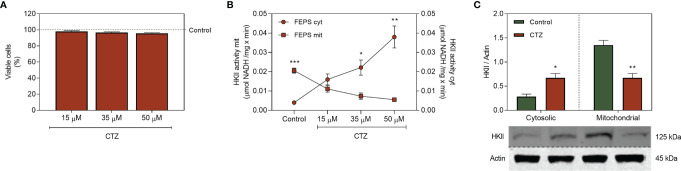
Effect of clotrimazole for the expression and activity of HKII on the mitochondrial and cytosolic fractions. FEPS cells were cultured as described in Materials and Methods. A total of 2×10^4^ cells were used to the viability assay, 60 µg of protein was used for enzymatic activity assay, and 40 µg of protein was used to Western blotting. **(A)** Percentages of viable cells were assessed by crystal violet after 1 h of incubation with 15 µM, 35 µM, and 50 µM CTZ. Data represent the mean percentage of viable cells + SEM relative to untreated control (dotted green line). **(B)** HKII activity measured through the conversion of NAD^+^ to NADH by G6PDH, considering the stoichiometric ratio where 1 mol of G6P produced is equivalent to 1 mol of reduced NAD^+^ in µmol NADH/mg × min after 1 h of incubation with 15 µM, 35 µM and 50 µM CTZ. **(C)** Expression of HKII was evaluated by Western blotting as described in Materials and Methods. Densitometric analyses were calculated in relation to the expression of actin for each cell under control conditions and after 1 hour of incubation with 35 µM CTZ. Data represent the mean + SEM of five independent experiments. Cytoplasmic HKII activity (red circle, FEPS cyt), Mitochondrial HKII activity (red square, FEPS mit), control FEPS (green bar), CTZ treatment (red bar). (*) p < 0.05, (**) p < 0.01, (***) p < 0.001.

CTZ is a synthetic antimycotic agent of the azole class which induces detachment of HK from the outer mitochondrial membrane (30–32). To discriminate the activity of HKII coupled to mitochondria from the one in the cytosolic fraction, FEPS was incubated for 1 h with CTZ, and a sub-toxic concentration of 35 μM was selected for the following experiments (**Figure 5A**). Remarkably, CTZ showed no cytotoxic effect towards the parental cell line K562, possibly reflecting the lower expression of mitochondrial HKII (**Supplementary Figure 1**). FEPS showed a significant dose-dependent increase in cytosolic HKII activity after CTZ treatment, while the activity on the mitochondrial fraction was correspondingly reduced (**Figure 5B**), reversing the profile previously observed (**Figure 3C**). This profile was not observed in K562 sensitive cells (**Supplementary Figure 2**). The increase in cytosolic HKII activity was accompanied by an increase in the cytosolic expression as well. Likewise, the reduction in mitochondrial activity was followed by a reduction in mitochondrial HKII expression (**Figure 5C**). This shift corroborates earlier observations indicating CTZ as an interesting approach to detach HKII from mitochondria.

### 3.6 Pharmacological Detachment of HKII From Mitochondria Alters Mitochondrial Parameters, Lactate Release and ATP Content in MDR Cells

**Figure 6:**
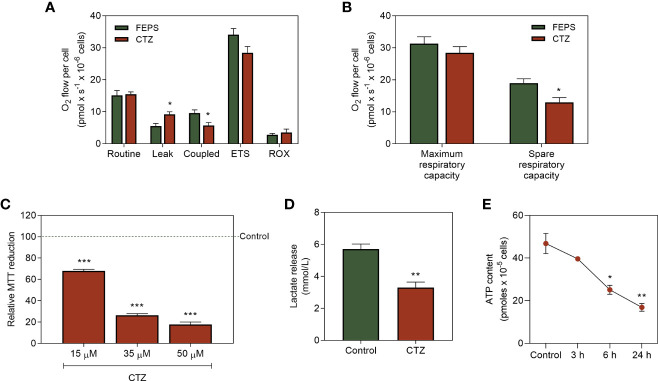
Effect of HKII detachment from mitochondria on the metabolic parameters of MDR CML cells. FEPS cells were cultured as described in Materials and Methods. A total of 2×10^6^ cells were used to high respirometer resolution, 2×10^6^ cells were used to MTT assay, 4×10^5^ cells were used to quantify lactate release, and a total of 10^5^ cells was used to quantify ATP content. **(A)** Oxygen flow in response to modulation of the ETS with 1 µg/mL of the complex I inhibitor oligomycin (leak), 50 nM of the proton ionophore FCCP (maximal respiratory capacity), 0.5 µM rotenone and 2 µg/mL antimycin A, respectively complex III and IV inhibitors (ROX). Data represent the mean oxygen flux + SEM in pmol normalized to the total cell count of control or cells incubated with 35 mM CTZ for 1 h. **(B)** Oxygen flow refers to the maximum and spare respiratory capacities of control FEPS and after incubation with 35 mM CTZ for 1 h, represented by the mean oxygen flow + SEM in pmol normalized by total cell count. **(C)** Evaluation of mitochondrial function by MTT reduction after incubation with 15 µM, 35 µM and 50 µM CTZ for 1 h. Data normalized to the control. **(D)** Indirect quantification of lactate released after 24 h through the formation of NAD^+^ from NADH under control conditions and after 1 h of 35 µM CTZ incubation. Data express the mean lactate release + SEM in mmol/L. **(E)** ATP content in accordance with the light emitted after reaction with luciferase under control conditions and after 3, 6 and 24 h with 35 µM of CTZ incubation. Data express ATP quantification mean + SEM in pmol. Representative data from three independent experiments. Control FEPS cells (green bars), FEPS incubated with CTZ (red bars). (*) p < 0.05, (**) p < 0.01, (***) p < 0.001.

The factors that regulate the proportions of HK that must be coupled to the outer mitochondrial membrane or free in the cytosol are still controversial in the literature. However, it is plausible that interferences in this balance would reflect directly on cellular metabolism. **Figure 6A** shows that the reduced mitochondrial expression of HKII leads to increased ATP synthesis-uncoupled oxygen flow (leak). This parallels with a decrease in the ATP synthesis-coupled oxygen flow. Though CTZ does not affect the maximum capacity, the spare respiratory capacity (**Figure 6B**) and mitochondrial function (**Figure 6C**) were significantly reduced upon incubation with CTZ. Interestingly, despite the increased cytosolic HKII activity (**Figure 5B**), both the lactate release (**Figure 6D**) and ATP content (**Figure 6E**) were considerably reduced after a 6 h incubation with CTZ. These results show that cytosolic translocation of HKII markedly disturbs the metabolic profile of chemoresistant CML cells.

### 3.7 Mitochondrial Membrane-Bound HKII Modulates the Antioxidant Protection in MDR Cells

**Figure 7:**
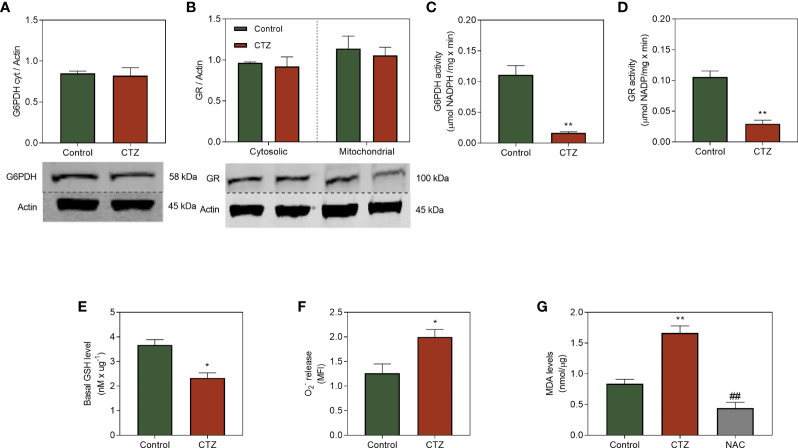
Effect of HKII detachment from mitochondria on the antioxidative system of MDR CML cells. FEPS cells were cultured as described in Materials and Methods. 40 µg of protein was used for western blotting, 60 µg and 100 µg of protein was used to G6PDH and GR enzymatic activity respectively, a total of 10^5^ cells was used to measure ${\text{O}}_{2}^{-}$ release, and 10^6^ cells was used to measure MDA levels. **(A, B)** Western blot protein quantification and densitometry analysis using anti-G6PDH and anti-GR antibodies and primary anti-actin antibodies. Densitometric analyses were calculated in relation to the expression of actin for each cell under control conditions and after 1 h of incubation with 35 µM CTZ. Data represent the mean + SEM of three independent experiments. **(C)** G6PDH activity was measured through the conversion of NADP^+^ to NADPH resulting from the oxidation of G6P to 6-phosphoglucone-δ-lactone, considering the stoichiometric ratio of 1 mol of 6-phosphoglycon-δ-lactone to 1 mol of NADPH as quantified by absorbance (340 nm), under control conditions and after 1 h with CTZ incubation. Data represent the mean of G6PDH activity + SEM in µmol NADPH/mg × min. **(D)** GR activity was measured through the reduction of oxidized glutathione molecule (GSSG) to GSH, using NADPH as an electron acceptor and converting it to NADP^+^. **(E)** Quantification of GSH levels through the reduction of oxidized glutathione molecule (GSSG) to GSH by GR after 1 h of CTZ treatment. Data represent the mean GSH content + SEM in nM/µg. **(F)** Superoxide release was quantified by MitoSOX under control conditions and after 1 h of CTZ incubation. Data represent the mean fluorescence intensity + SEM produced after the oxidation of the reagent by mitochondrial ${\text{O}}_{2}^{-}$ **(G)** MDA production after the reaction of free radicals with the mitochondrial membrane under control conditions, after 1 h of CTZ incubation, and after NAC incubation. Data represent the mean MDA + SEM released in nmol/µg of protein. Representative data from three independent experiments. Control FEPS (green bars), FEPS incubated with CTZ (red bars), FEPS after incubation with NAC (grey bar). (*) p < 0.05; (**) and (##) p < 0.01. (*) statistical significance compared to control; (#) statistical significance in relation to CTZ incubation.

The presence of an antioxidant system able to prevent cell damage generated by ROS is essential for adaptation to chemotherapeutic stress. CTZ did not affect G6PDH and GR quantification (**Figures 7A, B**), two critical enzymes for the antioxidative machinery. Conversely, the activity of those enzymes were drastically reduced (**Figures 7C, D**). However, this reduction was not caused by direct effect of CTZ, given that the activity of both were similar when the fractions were treated with this drug (**Supplementary Figure 3**). Equilibrium of glutathione between the reduced (GSH) or oxidized (GSSG) forms vary in accordance with the oxidizing stimuli which cells are exposed to. GR is responsible for maintaining the redox status of GSH by recycling the oxidized form, in a way that a decrease in its activity result in low levels of GSH. **Figure 7E** shows that FEPS presented low GSH content, which relates to the superoxide levels (**Figure 7F**) and mitochondrial damage (**Figure 7G**). This effect is not observed in K562, since pre-incubation with CTZ did not reduce G6PDH activity and did not increase lipid peroxidation levels in this cell line (**Supplementary Figure 4**).

### 3.8 The Metabolic Imbalance Caused by Translocation of HKII Synergizes With Chemotherapeutic Drugs and Increases Apoptotic Cell Death

**Figure 8:**
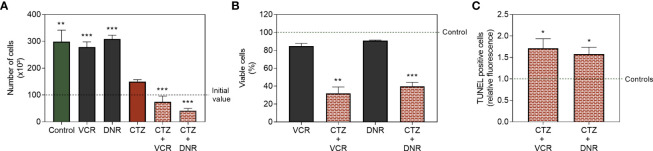
Cytotoxicity of standard chemotherapeutic drugs after detachment of HKII from mitochondria. FEPS cells were cultured as described in Materials and Methods. A total of 2×10^4^ cells was used for viability assay, and 2×10^5^ cells were used for TUNEL assay. **(A)** Trypan blue count of control and cells treated with 60 nM VCR and 500 nM DNR for 72 h; 35 µM CTZ followed by incubation with 60 nM VCR or 500 nM DNR for 72 h. **(B)** Viability of MDR cells treated in the same protocol measured by crystal violet, normalized to the untreated controls. **(C)** TUNEL-positive cells treated with 60 nM VCR or 500 nM DNR for 72 h following 1 h of treatment with 35 µM CTZ. Bars represent the mean values + SEM for each assay, representative of three independent experiments. Untreated FEPS (green bar, control), FEPS treated with CTZ (red bar), FEPS treated with VCR or DNR (black bars), FEPS treated with CTZ followed by VCR or DNR (red hatched bars). (*) statistical significance compared to initial value; (*) p < 0.05, (**) p < 0.01, (***) p < 0.001.

As demonstrated before, 1 h treatment with CTZ at 35 μM induced no evident cytotoxicity (**Figure 5A**). On the other hand, CTZ reduced FEPS proliferation by ~50% after 72 h, as shown in **Figure 8A**. Exposure to CTZ followed by treatment with the chemotherapeutic drugs VCR or DNR for 72 h further reduced cell viability (**Figures 8A, B**). As seen in **Figure 8C**, pre-incubation with CTZ caused collateral sensitization on FEPS cells, resulting in apoptotic death, as it increases the percentage of TUNEL-positive cells. These data corroborate reports that point to protection against apoptosis as one of HKII moonlight functions when attached to the mitochondria (16, 17).

### 3.9 Detachment of HKII From the Mitochondrial Membrane Modulates ABCC1 Activity

**Figure 9:**
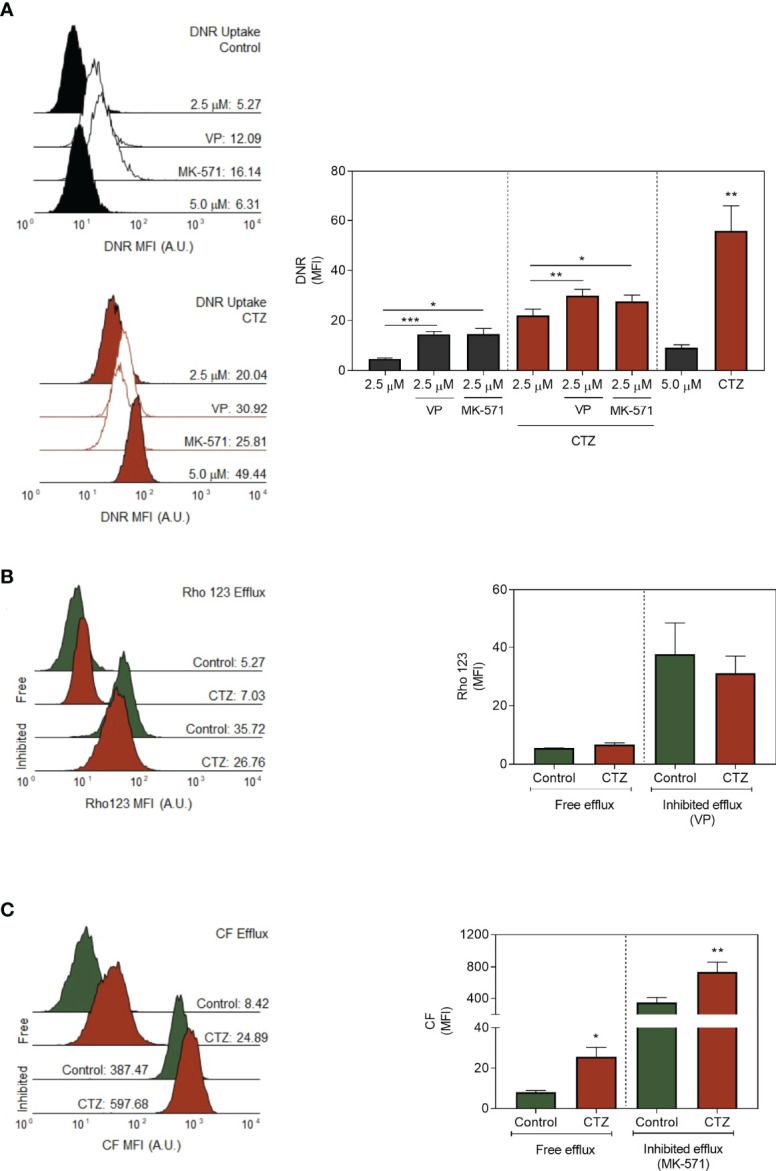
Profiles of ABC-mediated transport after CTZ treatment. **(A)** Effect of ABC inhibition for daunorubicin uptake. FEPS cells were treated with 35 µM CTZ for 1 h, then incubated in fresh medium containing 2.5 or 5.0 µM DNR for 1 h more in the absence or presence of 10 µM VP or 25 mM MK-571, respectively inhibitors for ABCB1 and ABCC-mediated transport. The MFI accounting for intracellular DNR was further acquired by flow cytometry. Black or red histograms indicate, respectively, untreated or CTZ-incubated cells. Controls (CTR) were treated with diluent. The MFI for whole populations is present on the right. Bars indicate the mean MFI + SEM for DNR obtained after 1 h. Black bars respectively indicate FEPS untreated cells, and red ones, CTZ treatment. n=6. **(B, C)** The transport activities by ABCC or ABCB1 were evaluated by Rho 123 or CF efflux assays. FEPS cells were treated with 35 µM CTZ for 1 h, then incubated in medium containing 250 nM Rho 123 or 500 nM CFDA for 30 min. Fresh media was added in the absence or presence of 10 µM VP or 25 mM MK-571, respectively inhibitors for ABCB1 and ABCC-mediated transport, for another 30 min. After incubation, the MFI accounting for intracellular Rho 123 or CF was acquired by flow cytometry. Representative histograms for ABCB1 **(B)** or ABCC1 **(C)** were divided into two areas: Rho 123- or CF-negative on the left and Rho 123- or CF-positive on the right, as described under Material and Methods. Green or red histograms indicate cells, respectively, in absence (free efflux) and in the presence of VP (inhibited efflux). Controls (CTR) were treated with diluent. The MFI for whole populations is present on the right. Bars indicate the mean MFI + SEM obtained for Rho 123 **(B)** or CF **(C)** after the free or inhibited Rho 123 or CF efflux, respectively. Green bars respectively indicate FEPS untreated cells, and red ones, CTZ treatment. n = 3 (ABCB1) or n = 5 (ABCC). (*) p < 0.05, (**) p < 0.01, (***) p < 0.001.

Among the mechanisms enhanced during adaptation to stress, the roles of ABC transporters are the most discussed. ABCB1 and ABCC1 promote the MDR phenotype on a variety of cancer models, including the FEPS cell line (2, 26, 27). In this context, the impact of CTZ on the functional activity of both transporters was evaluated. Corroborating the results in **Figure 8**, CTZ promoted an increase in DNR uptake, most noticeably when FEPS was incubated with 5.0 μM of this drug. This profile manifested in the presence of the ABCB1 and ABCC1 inhibitors, VP and MK-571, respectively, which further increased DNR uptake in co-treatment with CTZ (**Figure 9A**). A distinct scenario occurred when the functional assays were performed by use of specific substrates for each ABC protein. **Figure 9B** showed that the fluorescence of Rho 123 was not altered after CTZ treatment, whereas the one for CF increased in absence (free efflux) and in presence of MK-571 (inhibited efflux) (**Figure 9C**). Thus, detachment of HKII from the outer mitochondrial membrane impaired ABCC1 activity with no effect on ABCB1, allowing for increased DNR retention in FEPS.

### 3.10 GSH Partially Restores ABCC1 Activity Following the Metabolic Imbalance Triggered by the Translocation of HKII

**Figure 10:**
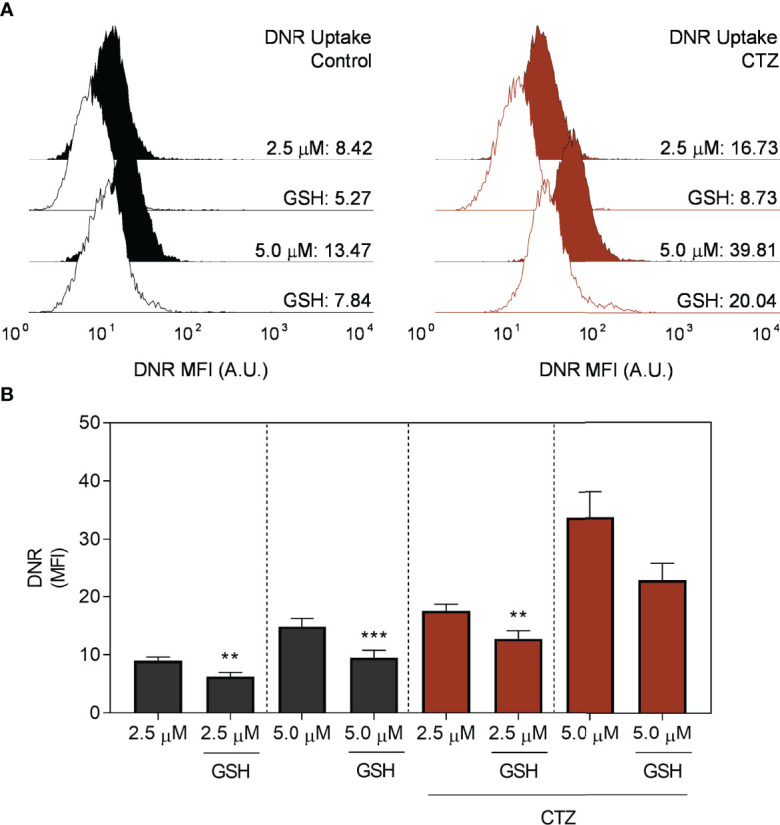
Effect of glutathione for daunorubicin uptake. FEPS cells were treated with 35 µM CTZ for 1 h, then incubated in fresh medium containing 2.5 or 5.0 µM DNR for 1 h more in the absence of presence of 5.0 mM GSH. The MFI accounting for intracellular DNR was then acquired by flow cytometry. **(A)** Black or red histograms indicate, respectively, untreated or CTZ-treated cells. Controls (CTR) were treated with diluent. **(B)** MFI for whole populations. Bars indicate the mean MFI + SEM for DNR obtained after 1 h. Gray bars respectively indicate FEPS untreated cells, and red ones, CTZ treatment with the addition of GSH where indicated. n=6. (**) p < 0.01, (***) p < 0.001.

A crucial feature of the ABCC-mediated transport is the interaction with GSH. Transport of the majority of ABCC1 substrates requires the presence of GSH, either in conjugation or in cotransport (35). Based on this, we evaluated whether GSH could impact the inhibitory effect of CTZ towards DNR efflux in FEPS (**Figures 10A, B**). Of note, co-treatment of CTZ and GSH reduced the accumulation of this drug to similar levels to the untreated controls. Considering that the cytosolic localization of HKII reduced the levels of this antioxidant peptide (**Figure 7E**) it is plausible to assume that GSH would antagonize the metabolic consequences of CTZ treatment. The negative modulation of ABCC1 activity (**Figure 9C**) could be a consequence of this effect since GSH is a prime substrate for this transporter.

## 4 Discussion

Metabolic reprogramming is well recognized as a hallmark of neoplastic growth, when cells undergoing malignant transformation resort to glycolysis to maintain a steady supply of ATP to support cell proliferation (43). Cells subjected to chemotherapeutic stress often develop similar yet specific changes in their energy metabolism that interplay with classical mechanisms that regulate the acquisition of chemoresistance (44).

This study employed an MDR cell line that requires a high energy supply promoted by the glycolytic pathway since the increased lactate production is linked to the high HKII expression. As such, the mitochondria of these cells consume less oxygen in favor of energy production, culminating in low respirometry rates when compared to the parental, non-resistant cell line. This pattern has already been reported in the literature in a model of lung cells resistant to doxorubicin, which consume less oxygen than their drug-sensitive counterpart (45). In addition, Nelson and colleagues argued that mitochondria from a model of acute myeloid leukemia undergo several metabolic changes resulting in increased aggressiveness (46). These alterations are directly linked to the use of electrons by the mitochondrial oxidative phosphorylation system (OXPHOS) for the production of mitochondrial ATP (46). Both studies demonstrated that low oxygen consumption is associated with increased mobilization of the glycolytic pathway in more aggressive cell lines (45, 46).

Previous studies show that chemoresistant cells have a high glycolytic rate along with overexpression of HKII (47–49). Upon inhibition of HKII, osteosarcoma cells reduce glycolysis and increase ATP synthesis through the consumption of mitochondrial oxygen. This suggests that HKII may regulate the metabolic shift that leads to an increase in glycolysis (49). Unlike these works, our group showed a high expression and activity of this enzyme in its mitochondria-bound form in MDR cells. This enzyme, when coupled to the mitochondrial membrane, would act as a moonlighting protein. According to this proposal, HKII could perform alternate functions to that of catalysis in metabolic reactions (17, 18). By directly consuming mitochondrial ATP, HKII makes the transport of electrons, through the ETS complexes, more efficient, reducing the probability that unpaired electrons would react with oxygen from the mitochondrial matrix and produce ROS (21, 50). Another critical feature of mitochondrial HKII is its interaction with VDAC, which interferes with the binding of Bax to this anion channel, thus compromising the apoptotic pathway (22, 23). Canonically, glucose phosphorylated by HK proceeds through the PPP (16) producing NADPH, which participates in the formation of reduced glutathione and a reducing coenzyme in other biosynthetic metabolic pathways, for example, during fatty acid biosynthesis. The primary role of GSH is to neutralize ROS, xenobiotics, and free radicals (51). In this sense, Catanzaro *et al.* demonstrated that MDR cell lines showed high activity of the first enzyme of the PPP, G6PDH (52). In line with the literature, our MDR model showed high G6PDH activity and antioxidant protection, maintaining low levels of ROS and MDA. Unlike K562, in which more than 75% of cells were viable within 24 h of treatment with 2DG, only 30% of FEPS remained viable in the same schedule, suggesting a greater degree of dependence on glycolysis and the PPP. This result is similar to other cell lines resistant to doxorubicin (53) or Adriamycin^®^ (48), which share structural and functional similarities to DNR (47). Therefore, HKII establish a link between the development of the MDR phenotype and mitochondria, along with the characteristics triggered by this interaction. Considering the importance of a competent antioxidative system to mitigate ROS damage, we propose that an imbalance in HKII activity would translate into a specific sensitization of MDR cells, a phenomenon known as collateral sensitivity.

Thus, understanding the metabolism of chemoresistant cells when HKII is not attached to the outer mitochondrial membrane is an excellent attempt to unravel the relationship of this protein with the MDR phenotype. CTZ is an antifungal compound described as a calmodulin agonist, capable of interfering with the metabolism of Ca^+2^ and with K^+^ channels, causing instability in the mitochondrial membrane and, consequently, separating HKII from the VDAC (30–32). In this line, Penso and Beitner established a protocol where the incubation with CTZ decreased mitochondrial-bound HKII levels with a concomitant increase in the soluble fraction in melanoma cells (32). Following the CTZ pre-incubation, in our model, both the enzymatic activity and the protein expression of HKII were increased in the soluble fraction, along with a decrease in oxygen consumption coupled with ATP synthesis, and higher proton leakage through the inner mitochondrial membrane. The reduced effects, or lack thereof, of CTZ on K562 cells (with low HK expression) support the proposal that the drug resistance is associated with the high HK expression in FEPS cells because CTZ did not modify the mitochondrial parameters and glycolysis in K562. As such, the effects induced in these cells might be attributed to other causes not associated with the removal of HKII from the outer mitochondrial membrane. Decreased expression and activity of mitochondrial HKII in FEPS did not change the basal oxygen flow profile, indicating that the mitochondria no longer efficiently use oxygen for OXPHOS, thus reducing its spare capacity. This suggests that mitochondria from MDR cells likely lose their adaptability power with the altered expression of HKII, which may as well justify the reduction in mitochondrial function after FEPS was incubated with CTZ. This, however, does not exclude the possibility of CTZ causing the loss of the mitochondrial function apart from the re-localization of HKII. Several studies show that CTZ directly affects cell glycolysis by inhibiting key enzymes and, consequently, disrupting ATP synthesis (54, 55). In this work we observed lactate release and a decrease of ATP content following CTZ treatment, though not a decline in HKII enzymatic activity, even with an increase in activity in the cytosolic fraction. However, it may be necessary to evaluate the activity of other glycolytic enzymes to confirm whether this profile is consistently repeated in FEPS.

Increased glycolysis, however, would probably not be the sole responsible for the higher ATP content observed in MDR cells. The selective pressure exerted by chemotherapeutic drugs often target central cellular mechanisms, resulting in reduced replicative fitness (56). Previous works from our group showed that FEPS present higher expression of genes associated to a quiescent, stem-like phenotype such as *Notch* (25), *Oct-1*, *Sox-2* and *MDR1* (57) when compared to the parental K562. Moreover, FEPS present reduced proliferation rate than K562, with a higher percentage of cells in the G1/S phase after 96 h, and silencing ABCB1 did not alter the dynamics of cell cycle (26). In MDR cells, diverse mechanisms work in tandem to reduce stress; irrespective of increased drug efflux being one of the most studied of those along with metabolic reprogramming, it is expected that each adaptation during the acquisition of the MDR phenotype would reflect on the energy consumption and total ATP concentration.

In breast cancer cells, CTZ reduces the activity of G6PDH (54), as well as interferes with the cell’s glutathione levels, which favors the accumulation of ROS (58). These conditions may not occur in isolation since the HKII linked to mitochondrial VDAC participates in the production of intramitochondrial ROS, and its detachment from VDAC might increase oxidative stress (59, 60). Our data are in line with this premise, considering that the activity of G6PDH and GR are compromised after treatment with CTZ. Such commitment does not seem to be caused by any direct inhibitory effect of CTZ on these enzymes. When pre-incubation of K562 cell line with CTZ is performed, under the same conditions as FEPS, G6PDH activity was not compromised. As the parental cell line does not have high expression and activity of HKII, its detachment of the mitochondrial membrane may not occur at significant levels to the point of causing a bioenergetic modification in the cell. It is well established in the literature that HK, when bound to mitochondria, shows an increased ability to phosphorylate glucose since it is not inhibited by its product, G6P (38, 61). In addition, there is a crosstalk between mitochondrial HKII and PPP, since the production of G6P serves as subsidy for G6PDH to trigger its functions in the cell (62, 63). Redhu and Bhat also showed that, by interfering with the interaction between HK and mitochondria, G6PDH levels were reduced, raising mitochondrial ROS levels (63). Therefore, we propose that the removal of HK from the mitochondrial membrane would cause a decrease in the production of G6P, which, in turn, would not be available to enter the PPP on a large scale. Thus, the activity of G6PH would be impaired and, in consequence, the production of GSH by GR since this chain of events primarily depends on the G6PDH activity. The increase in damage to mitochondrial membrane lipids, caused by the reduction of GR activity, is significantly attenuated in the presence of NAC, as it acts both as a scavenger of ROS and as precursor to the synthesis of GSH (64). Furthermore, by reducing the activity of G6PDH and interfering with PPP, cell proliferation may be compromised since this pathway is important for the production of ribonucleotides that will be used in the process of cell division (16). Thus, CTZ is known to promote cell cycle arrest and to interfere with the bioenergetic metabolism of a number of cell lines (31, 65, 66).

A collection of works demonstrated a moonlighting role of HKII in protecting against apoptosis (67–70). By binding to mitochondrial VDAC, it blocks the passage of important proteins for the apoptotic pathway, such as Bax and Bak, consolidating its anti-apoptotic role (70). CTZ, in contrast, promotes the detachment of HKII from the mitochondrial membrane, which liberates the anion channel and increases apoptosis (67, 68). The multiple roles of HKII, linking the adaptation to stress to the regulation of energy metabolism, would offer an interesting opportunity to develop a treatment regimen directed to chemotherapy-refractory cancers. MDR is the main hurdle to treatment success, and the upregulation of ABCB1 or ABCC1 (26), should be taken into consideration for the success of such a strategy. Exploiting the proposed moonlighting role of HKII would lead to a phenomenon named collateral sensitivity (71, 72), which could re-sensitize MDR cells to standard drugs such as DNR or VCR. Conversely, when each ABC transporter was assessed with a combination of specific substrates and inhibitors, CTZ impaired the transport capacity only of ABCC1. This inhibition translated into higher accumulation of DNR, which is in accordance with the chemosensitization observed before. Our results propose that MDR cells overexpressing ABCC1 would readily counter oxidative stress by transporting GSSG to the extracellular milieu, relying on the higher ATP content. Treating FEPS with CTZ severely hampered these processes, increasing the release of ROS, reducing the activity of glutathione reductase, the availability of GSH and of ATP required for ABCC1-mediated efflux. As a consequence, DNR, or any cytotoxic xenobiotic or secondary metabolite that would be cleared by this transporter might accumulate inside the cell. There is a certain level of promiscuity and overlap in the recognition of substrates by ABC transporters (35), but despite DNR being transported both by ABCB1 and ABCC1, results indicate that the first is not affected by the impairment of mitochondrial HKII caused by CTZ. As active transporters, both engage in constant ATPase activity, but if only one of the transporters is affected by the decreased expression of mitochondrial HKII, then the reduction in ATP content may not be the preponderant factor. The direct interaction between CTZ and ABC transporters is still controversial in the literature. Few observations suggest that CTZ indirectly inhibits ABCB1, interacting with sites that do not entirely prevent its efflux capacity (73–75); considering ABCC1, CTZ would inhibit its activity, but without a clear-cut mechanism by which this would occur (76, 77).

One of the features that differentiate ABCC1-mediated efflux from the one of ABCB1 is the participation of GSH that favors the transport of xenobiotics either in conjugation to or in cotransport with it (78). Some groups showed that ABCC1 has an affinity for GSH, which would help transport some chemotherapeutic drugs, but the physicochemical properties of this relationship remain unclear (79, 80). Considering that the detachment of HKII from the mitochondria caused an imbalance in the antioxidant metabolism of FEPS and the addition of exogenous GSH restored the ABCC1-mediated efflux reducing DNR uptake, our proposal for the sensitization seems reasonable.

**Figure 11:**
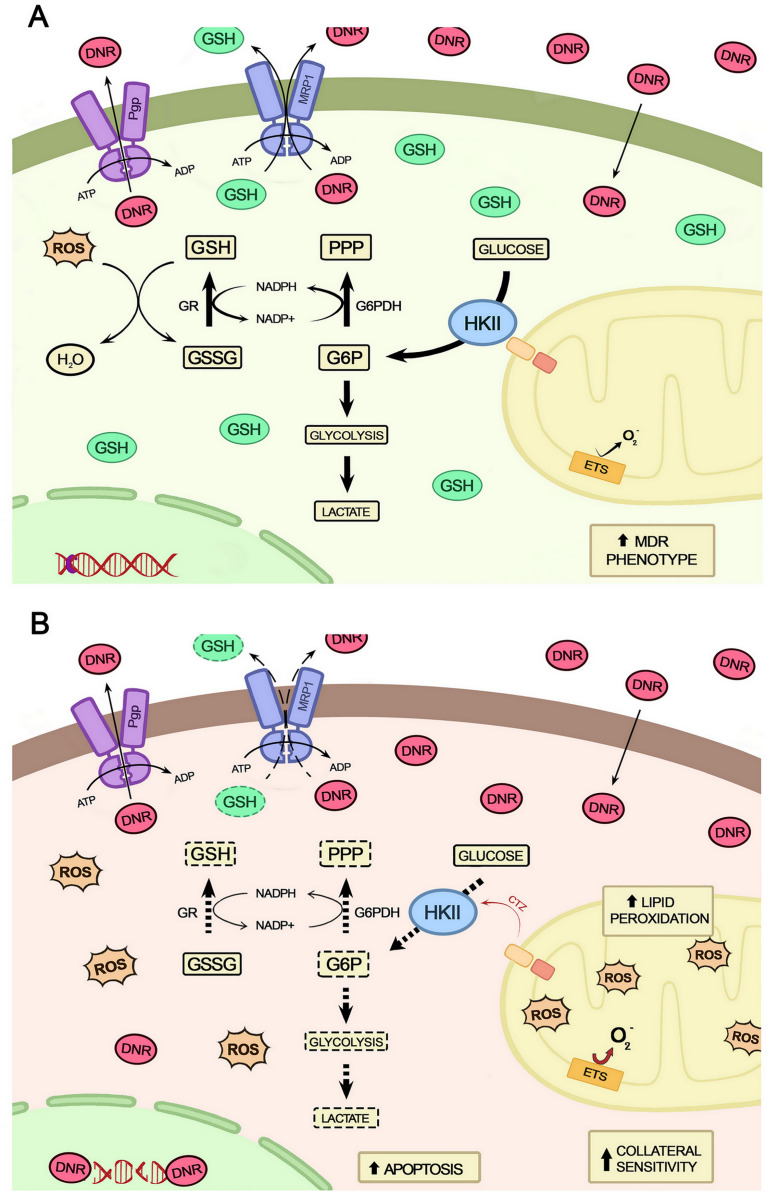
Schematic representation of the relationship between the detachment of HKII from mitochondria and collateral sensitivity. **(A)** Cells with MDR phenotype mobilize a complex architecture to deal with the chemotherapeutic and oxidative stresses by increasing their ATP output, the activity of ABC transporters, and the production of antioxidant molecules. **(B)** When HKII detaches from mitochondria, this architecture is destabilized, resulting in impairment of metabolic pathways for energy generation (glycolysis and OXPHOS), reduced antioxidative capacity by consumption of GSH, and diminished ABCC1 activity, ultimately leading cells to increased drug uptake and to apoptotic death.

In conclusion, the data obtained in this study indicate a central role for the mitochondrial HKII in regulating energy metabolism to maintain the bioenergetic and antioxidant homeostasis of the MDR cells (**Figure 11**). Thus, such functions added to the moonlighting characteristics of mitochondrial HKII can strongly contribute to the maintenance of all the metabolic architecture necessary for the MDR phenotype. As such, this complex framework would likely drive innovation in the treatment of chemotherapy-refractory cancers.

## Data Availability Statement

The raw data supporting the conclusions of this article will be made available by the authors, without undue reservation.

## Author Contributions

TO, DL, LJ, JK, VA and ES performed the experiments. TO and DL wrote original draft and contributed equally then should be considered as first authors. VR developed the MDR CML model used in this work. RM coordinated the project. ES, VR and RM revised the manuscript. All authors contributed to the article and approved the submitted version.

## Funding

Conselho Nacional de Desenvolvimento Científico e Tecnológico (CNPq) (PQ/2018 CNPq 309946/2018-2), Fundação de Amparo à Pesquisa do Estado do Rio de Janeiro (FAPERJ) (Faperj CNE 2018 E-26/202.871/2018 and Faperj Sediadas 2018 E-26/010.101035/2018), and Coordenação de Aperfeiçoamento de Pessoal de Nível Superior (CAPES) (CAPES c/ grant 23038.009431/2021-42) supported this investigation.

## Conflict of Interest

The authors declare that the research was conducted in the absence of any commercial or financial relationships that could be construed as a potential conflict of interest.

## Publisher’s Note

All claims expressed in this article are solely those of the authors and do not necessarily represent those of their affiliated organizations, or those of the publisher, the editors and the reviewers. Any product that may be evaluated in this article, or claim that may be made by its manufacturer, is not guaranteed or endorsed by the publisher.

## Glossary

**Table:**

2DG	2-deoxy-D-glucose
6PG	6-phosphogluconate
6PDGH	6-phosphogluconate dehydrogenase
ATP	adenosine triphosphate
BSA	bovine serum albumin
CF	carboxyfluorescein
CFDA	5 (6)-carboxyfluorescein diacetate
CML	chronic myeloid leukemia
CTZ	clotrimazole
DMSO	dimethylsulfoxide
DNR	daunorubicin
DTT	1,4-dithiothreitol
EDTA	ethylenediamine tetraacetic acid
ETS	electron transport system
FCCP	carbonyl cyanide 4-(trifluoromethoxy)phenylhydrazone
FBS	fetal bovine serum
G6P	glucose-6-phosphate
G6PDH	glucose-6-phosphate dehydrogenase
GR	glutathione reductase
GSH	glutathione reduced form
GSSG	glutathione oxidized form
HK	hexokinase
LDH	lactate dehydrogenase
NaF	sodium fluoride
MDA	malondialdehyde
MDR	multidrug resistance
MgCl_2_	magnesium chloride
MK-571	5-(3-(2-(7-chloroquinolin-2-yl)ethenyl)phenyl)-8-dimethylcarbamyl-4,6-dithiaoctanoic acid sodium salt hydrate
MRP1/ABCC1	multidrug resistance-associated protein 1/ATP binding cassette subfamily C member 1
MFI	median fluorescence intensity
MTT	3-(4,5-dimethylthiazol-2-yl)-2,5-diphenyltetrazolium bromide
NAC	N-acetyl-L-cysteine
NAD	nicotinamide adenine dinucleotide
NADP	nicotinamide adenine dinucleotide phosphate
OXPHOS	mitochondrial oxidative phosphorylation system
PBS	phosphate buffered saline
Pgp/ABCB1	P-glycoprotein/ATP binding cassette subfamily B member 1
PPP	pentose phosphate pathway
qPCR	real time quantitative polymerase chain reaction
Rho 123	rhodamine 123
ROS	reactive oxygen species
SDS	sodium dodecyl sulfate
TBA	thiobarbituric acid
TBS	tris-buffered saline
TCA	trichloroacetic acid
TdT	deoxynucleotidyl transferase
TMP	tetramethoxypropane
UTP	uridine triphosphate
VCR	vincristine
VDAC	voltage-dependent anion channel
VP	verapamil
