# Supplementary material for: Detachment of Hexokinase II From Mitochondria Promotes Collateral Sensitivity in Multidrug Resistant Chronic Myeloid Leukemia Cells
Source: Front Oncol. 2022 May 26;12:852985. doi: 10.3389/fonc.2022.852985 (PMC9204307; doi:10.3389/fonc.2022.852985)
Supplement: Supplementary file 1 [file DataSheet_1.pdf]

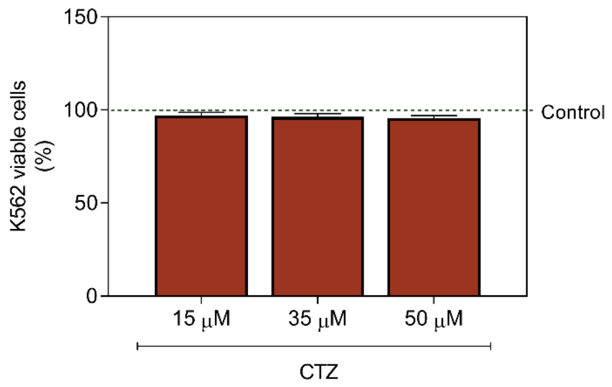

Supplementary Figure 1. Effect of clotrimazole on the viability of sensitive CML cells. K562 cells were cultured as described in Materials and Methods. A total of 2×10^4 cells were used to the viability assay. Percentages of viable cells were assessed by crystal violet after 1 h of incubation with 15 µM, 35 µM, and 50 µM CTZ. Data represent the mean percentage of viable cells + SEM relative to untreated control. Data represent the mean + SEM of five independent experiments.

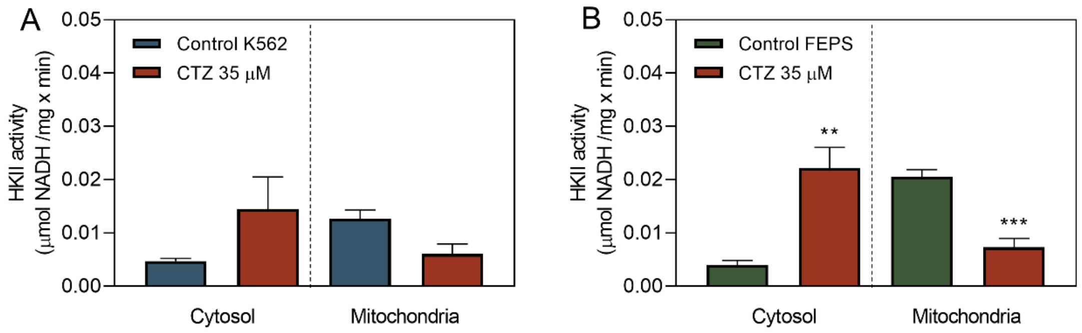

Supplementary Figure 2. Effect of clotrimazole for the activity of HKII on the mitochondrial and cytosolic fractions. K562 and FEPS cells were cultured as described in Materials and Methods. 60 µg of protein was used for enzymatic activity assay. HKII activity measured through the conversion of NAD^+ to NADH by G6PDH, considering the stoichiometric ratio where 1 mol of G6P produced is equivalent to 1 mol of reduced NAD^+ in µmol NADH/mg × min after 1 h of cells incubation with 35 µM CTZ. **(A)** HKII activity in K562 cytosolic and mitochondrial protein extracts of K562 control or treated with 35 µM CTZ after 1 h. **(B)** HKII activity in the cytosolic and mitochondrial protein extracts of FEPS control or treated with 35 µM CTZ after 1 h. Data represent the mean + SEM of three independent experiments. K562 (blue bars), FEPS (green bars), CTZ treatment (red bars). (**) p<0.01, (***) p<0.001.

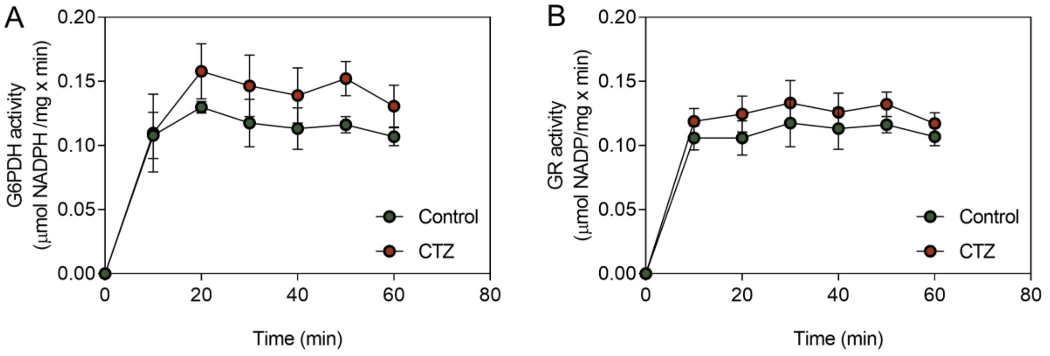

Supplementary Figure 3. G6PDH and GR enzymatic activity after direct clotrimazole treatment on the mitochondrial and cytosolic fractions. FEPS cells were cultured as described in Materials and Methods. 60 µg and 100 µg of cytosolic protein were respectively used to the enzymatic activities. G6PDH and GR activities were quantified by absorbance (340 nm), under control conditions and up to 1 h following CTZ treatment of the cytosolic and mitochondrial fractions. **(A)** G6PDH activity was measured through the conversion of $NADP^+$ to NADPH resulting from the oxidation of G6P to 6-phosphoglucone-δ-lactone, considering the stoichiometric ratio of 1 mol of 6-phosphoglycon-δ-lactone to 1 mol of NADPH. Data represent the mean of G6PDH activity + SEM in µmol NADPH/mg × min without the 6PGDH activity contribution as described in Material and Methods. **(B)** GR activity was measured through the reduction of oxidized glutathione molecule (GSSG) to GSH, using NADPH as an electron acceptor and converting it to $NADP^+$. Data represent the mean of GR activity + SEM released in µmol NADP/mg × min nmol/µg of protein. Representative data from three independent experiments. Control FEPS (green circles), FEPS incubated with CTZ (red circles).

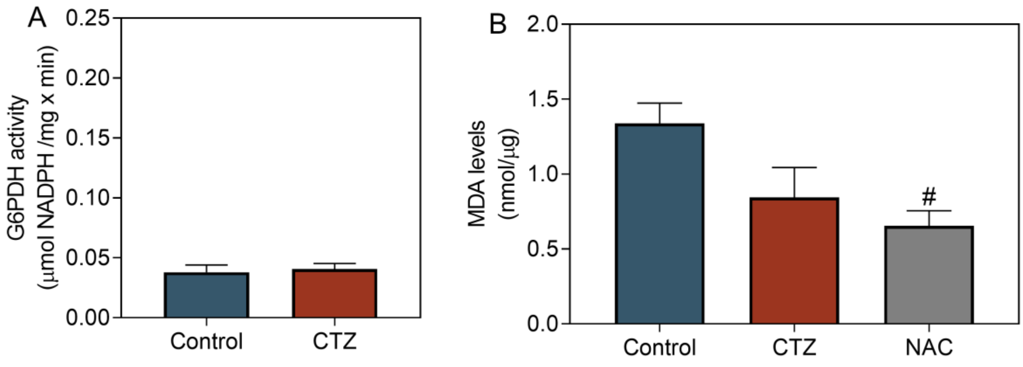

Supplementary Figure 4. Effect of HKII detachment from mitochondria on the antioxidative system of sensitive CML cells. K562 cells were cultured as described in Materials and Methods. 60 µg of protein was used to G6PDH enzymatic activity and 10^6 cells were used to measure MDA levels. **(A)** G6PDH activity measured through the conversion of $NADP^+$ to NADPH resulting from the oxidation of G6P to 6-phosphoglucone-δ-lactone, considering the stoichiometric ratio of 1 mol of 6-phosphoglycon-δ-lactone to 1 mol of NADPH as quantified by absorbance (340 nm), under control conditions and after 1 h with CTZ treatment. Data represent the mean of G6PDH activity + SEM in µmol NADPH/mg × min. **(B)** MDA production after the reaction of free radicals with the mitochondrial membrane under control conditions, after 1 h treatment with 35 µM CTZ, and after NAC incubation. Data represent the mean MDA + SEM released in nmol/µg of protein. Representative data from three independent experiments. Control K562 (blue bars), K562 incubated with CTZ (red bars), K562

after incubation with NAC (grey bar). (#) $p<0.05$; (#) statistical significance in relation to CTZ treatment.
